# A New Lectin from *Auricularia auricula* Inhibited the Proliferation of Lung Cancer Cells and Improved Pulmonary Flora

**DOI:** 10.1155/2021/5597135

**Published:** 2021-07-10

**Authors:** ZhenDong Liu, Liang Li, Bei Xue, DanDan Zhao, YanLong Zhang, XiuFeng Yan

**Affiliations:** ^1^Key Laboratory of Saline-Alkali Vegetation Ecology Restoration, Ministry of Education, College of Life Sciences, Northeast Forestry University, Harbin 150040, China; ^2^Food Science College, Tibet Agriculture & Animal Husbandry University, Nyingchi 860000, China; ^3^Sino-Russian Joint Laboratory of Bioactive Substance, College of Life Science, Heilongjiang University, 150080, China; ^4^College of Life and Environmental Science, Wenzhou University, Chashan University Town, Wenzhou 325035, China

## Abstract

Lectins are widely distributed in the natural world and are usually involved in antitumor activities. *Auricularia auricula* (*A. auricula*) is a medicinal and edible homologous fungus. *A. auricula* contains many active ingredients, such as polysaccharides, melanin, flavonoids, adenosine, sterols, alkaloids, and terpenes. In this study, we expected to isolate and purify lectin from *A. auricula*, determine the glycoside bond type and sugar-specific protein of *A. auricula* lectin (AAL), and finally, determine its antitumor activities. We used ammonium sulfate fractionation, ion exchange chromatography, and affinity chromatography to separate and purify lectin from *A. auricula*. The result was a 25 kDa AAL with a relative molecular mass of 18913.22. Protein identification results suggested that this lectin contained four peptide chains by comparing with the UniProt database. The FT-IR and *β*-elimination reaction demonstrated that the connection between the oligosaccharide and polypeptide of AAL was an N-glucoside bond. Analyses of its physical and chemical properties showed that AAL was a temperature-sensitive and acidic/alkaline-dependent glycoprotein. Additionally, the anticancer experiment manifested that AAL inhibited the proliferation of A549, and the IC_50_ value was 28.19 ± 1.92 *μ*g/mL. RNA sequencing dataset analyses detected that AAL may regulate the expression of *JUN*, *TLR4*, and *MYD88* to suppress tumor proliferation. Through the pulmonary flora analysis, the bacterial structure of each phylum in the lectin treatment group was more reasonable, and the colonization ability of the normal microflora was improved, indicating that lectin treatment could significantly improve the bacterial diversity characteristics.

## 1. Introduction

Lectins are proteins or glycoproteins that have at least one carbohydrate or derivative binding site and are different from immunoglobulin in nature and do not have the function of catalytic enzymes. They can specifically recognize and bind to sugars or sugar chains without changing the covalent structure [1]. Lectins are widely distributed in nature, ranging from microorganisms to animals and plants. It was named lectin because it can agglutinate blood cells and make the blood cells show reticular sedimentation [2]. Fungi lectins are the most studied in the past decades and show different structures, functions, and carbohydrate-binding specificities [3]. Lectins have usually been used as a tool to distinguish between cell types and have been involved in several biological activities such as mitogenic [4], anti-insect [5], anti-inflammatory [6], antimicrobial [7], and antitumor [8] activities. Undoubtedly, lectins can serve as a therapeutic goldmine in the near future. In the past decade, a flux of interest in the study of lectins from natural sources has been observed [9]. Fungi have not only turned into a rich hotspot for new lectins with extraordinary sugar specificities but have also turned into potential candidates for biomedical applications [10]. Many microfungal strains from *Fusarium* sp. [11–13] and *Penicillium* sp. [14–16] have been investigated for lectin activity. Various fungal lectins exhibit interesting physiological impacts such as mitogenic incitement of lymphocytes/splenocytes [17], suppression of cancer cell proliferation [18], and as immunomodulators [19]. In the study on the inhibition of tumor activity of lectin, Li et al. found that feeding mice *Pleurotus citrinopileatus* lectin at a dose of 5 mg/kg per day for 20 days could effectively inhibit the growth of 80% mouse sarcoma [20]. Li et al. found that purified *Hericium erinaceus* lectin with a molecular weight of 51 kDa could inhibit the proliferation of human liver cancer HepG2 and human breast cancer McF-7 cells [21]. Gondim et al. found that lectin in the seeds of a Brazilian fruit could inhibit the proliferation of human ovarian cancer A2780, lung cancer A549, breast cancer McF-7, and prostate cancer PC3 cells. In addition, lectin could block the retention of ovarian cancer cells in the G2/M phase, activate the expression of caspase 9, and delay cell apoptosis after 24 h of lectin action [22]. Chakkere et al. found that lectin in the fruit of Indian schistosomes could effectively inhibit the growth of human chronic myeloid leukemia K562, human colon cancer HT29, human cervical cancer HeLA, and human breast cancer McF-7 cells [23]. Lacerda et al. found that when the concentration of lectin was 100 mg/mL, it could inhibit 83% of mouse melanoma cells; lectin also had a protective effect on the stomach [24]. Liao et al. found that mussel lectin could bind to Gb3 on tumor cells and promote apoptosis of breast cancer cells [25].

*Auricularia auricula* (*A. auricula*) is a medicinal and edible homologous fungus [26]. In the biological classification system, *A. auricula* belongs to the fungus world basidiomycete. *A. auricula* contains many active ingredients, such as polysaccharides, melanin, flavonoids, adenosine, sterols, alkaloids, and terpenes, which can play a crucial role in the molecular recognition mechanism of cell-cell and cell-matrix interactions [27]. These macromolecules can reversibly bind to specific sugars and precipitate polysaccharides, glycoproteins, and glycolipids, so they can be used as cell recognition factors to participate in the targeting of tumor cells and have high medicinal value and antitumor activity. At an early stage, lectins in plants were studied deeply by scholars, most of which were about the seeds of leguminous plants. While for the fungus, Zhao et al. isolated two lectins with the same single subunit of 15.8 kDa from the fruiting body of *Agrocybe cylindracea*, and these lectins can bind to HeLa cells as a special signaling molecule and promote their apoptosis for the first time [28].

Lung cancer (LC) is one of the most dangerous malignancies with the fastest increase in morbidity and mortality. In the past 50 years, a significant increase in the incidence and mortality of lung cancer has been reported in many countries [29]. In recent years, LC is still one of the most common malignant tumors in human beings, and the incidence and mortality of LC are increasing year by year [30, 31]. There are two main types of lung cancer, namely, small-cell lung cancer (SCLC) and non-small-cell lung cancer (NSCLC) [32, 33]. Although researchers are beginning to develop therapeutic targets for LC, the prognosis of patients after treatment is poor, and the recurrence rate of patients after surgery is as high as 35-50% [34]. Therefore, it is essential to seek a novel therapy to improve the survival rate.

Therefore, this study was expected to isolate and purify lectin from *A. auricula*, determine the glycoside bond type and sugar-specific protein of *A. auricula* lectin (AAL), and finally determine its antitumor activities. By studying the biological activity of AAL and analyzing its ability to inhibit tumor proliferation, we can discover its important role in the research and development of clinical tumor drugs.

## 2. Materials and Methods

### 2.1. *A. auricula* and Lung Cancer Cell Lines

*A. auricula* was picked at the Nyingchi Prefecture, Millin County (Tibet, China), screening for the fruiting body that is large and full and with bright color for the test operation strain. A549 lung cancer cells were purchased from the Cell Bank of Shanghai Academy of Sciences.

### 2.2. Lectin Extract and Purification

#### 2.2.1. Lectin Extract

To isolate AAL, a fresh *A. auricula* was taken, and its surface moisture was sucked dry with filter paper. After being dried by a freeze dryer, 5 grams of dried *A. auricula* was accurately weighed, 400 mL of 10 mM phosphate buffer was added, and the mixture was stirred into a homogenate by a blender. The supernatant was taken and centrifuged for 20 minutes at 2795 × g for a blood agglutination activity test. After the homogenate of *A. auricula* was precipitated by 20%-80% ammonium sulfate for 12 h, it was placed in a refrigerator at 4°C for overnight magnetic stirring for dialysis; then, the solution in the dialysis bag was transferred in batches to a freeze-drying machine for lyophilization and labeling. Lyophilized powder was used for a hemagglutination activity test and protein quantitative analysis to determine the optimal saturation of lectin extraction. The dry weight, protein concentration, and agglutination activity of the product were analyzed.

#### 2.2.2. Lectin Purification

An AKTA Purifier100 protein purification system was selected for the purification of *A. auricula* lectin. 10 mM phosphate buffer solution and 20% ethanol solution were prepared in advance. The bubbles were removed by an ultrasonic cleaning machine for 10 min, then the AKTA system was connected. After the freeze-dried powder precipitated by ammonium sulfate was fully dissolved by phosphate buffer, the HiTrap DEAE Anion Exchange Column was prebalanced with phosphate buffer. When the samples attached to the DEAE column, phosphate buffer containing 0.1 M NaCl was used for elution at an elution rate of 0.5 mL/min and a system pressure of 0.3 MP. With the increase of the concentration of the eluent, the elution peak began to appear. According to the peak appearing time for collecting eluent, all the peak areas of the eluent were collected, and the active components of coagulation were determined by a hemagglutination test and then lyophilized in the freeze dryer after dialysis.

The dialysis freeze-dried powder was dissolved and combined with activated PSM-Sepharose in an ice bath for 2 h, the samples were placed in a refrigerated centrifuge and spun at1000 × g for 10 minutes, the supernatant was sucked out at a low temperature for preservation, PSM-Sepharose was cleaned three times by phosphate buffer and combined with 0.1 M of glycine at different pH levels (pH 1.5, pH 3.0, and pH 4.5) in an ice bath for 10 min, the samples were placed in a refrigerated centrifuge for 10 minutes and spun at1000 × g, and the supernatant was taken and the pH was adjusted to neutral; the Tris-HCl with a pH of 8.5 was used. Four ultrafiltration tubes were placed in a refrigerated centrifuge for 1 h, 3 h, and 5 h, respectively, and the solution after ultrafiltration was lyophilized to obtain lectin, which was precisely weighed and the extraction rate of lectin was calculated. The initial supernatant was detected by a hemagglutination test. If there was activity, the above process should be repeated until the lectin was fully bound to PSM-Sepharose. According to the Laemmli method [35], the purity of lectin was determined by SDS-polyacrylamide gel electrophoresis (SDS-PAGE).

#### 2.2.3. Structural Identification

The molecular weight of lectin was determined by SDS-PAGE, and the bands were obtained by ammonium sulfate precipitation, ion exchange chromatography, and affinity chromatography. The relative molecular mass of lectin can be accurately determined by using matrix-assisted laser desorption-ionization time-of-flight mass spectrometry (MALDI-TOF/TOF 5800 System) [36]. The 4000 Series Explorer V3.5 was used for data and graph processing.

The lectin was determined by a mass spectrometer (Applied Biosystems, MALDI-TOF/TOF 5800) for protein identification by primary and secondary mass spectrometry. Through UniProt database comparison, it was concluded that the lectin might contain peptides. The Edman degradation method is mainly used in the analysis of N-terminal amino acids. The lectins were first subjected to SDS-polyacrylamide gel electrophoresis, and the proteins in the gel were transferred to a PVDF membrane through an electric transfer slot. After setting by PPSQ-30 Analysis Software, N-terminal sequencing was performed on a PPSQ-30 automatic protein polypeptide sequencing instrument (Shimadzu). PPSQ-30 Data Processing Software was used for exporting data and graphs. The presence and the amount of lectin in the collected fractions were confirmed with the BCA Protein Quantification Kit (Pierce Biotechnology, Rockford, IL, USA). The sugar content of lectin was determined by the phenol-sulfuric acid method [37].

### 2.3. Analysis of Physicochemical Properties

#### 2.3.1. Effects of Temperature and pH on Lectin Activity

A solution of 1 mg lectin was prepared and incubated in a water bath at 20°C-100°C for 40 min, respectively, to observe the coagulation activity of the lectin. The ability of agglutinating rabbit blood was detected by dissolving lectin in PBS with different pH levels.

#### 2.3.2. Identifying the Sugar Specificity of Lectin

Lectins bind to sugars or proteins through sugar-binding sites (CRD), so lectins usually have specific binding sugars or proteins. After various sugars (derivatives) and proteins are added to the lectin solution, the inhibition of lectin coagulation activity is verified. Monosaccharides such as glucose, galactose, mannose, xylose, rhamnose, sialic acid, N-acetylgalactosamine, and pig gastric mucosa proteins were taken to prepare a sugar solution and a protein solution, respectively. The sugar and the protein solutions were diluted one by one, and then 30 *μ*L was drawn from each of them and added to the 96-well plates and the same volume of 2% rabbit blood was added. They were left at room temperature for 1 hour. We observed whether the sugar solution and the protein solution have any influence on the rabbit blood agglutination and can eliminate the influence of themselves on the blood coagulation activity. We take 0.1 g AAL, make up a 100 *μ*g/mL of lectin solution by using the PBS mixture, then draw 30 *μ*L in turn and add it to each hole of the 96-well plate. We then add 30 *μ*L of the concentration of the sugar solution and the protein solution, and we finally add 2% rabbit blood of the same volume, keeping the solutions at room temperature for 1 hour and then observing the clotting results.

### 2.4. Antitumor Activity Potential Evaluation of Lectin in A549 Cells

#### 2.4.1. A549 Cell Culture

A549 lung cancer cells were cultured in RPMI 1640 medium (90% 1640 medium + 10% FBS + 1% double antibody, i.e., a mixture of penicillin and streptomycin), and the resuscitation lung cancer cells (A549) were cultured for 2-3 generations. The antitumor experiment was carried out when the cancer cells were in the logarithmic growth stage.

#### 2.4.2. Cell Inhibition Analysis

The Cell Counting Kit-8 was used to detect the inhibition of lectin on the proliferation of tumor cells. Double distilled H_2_O was added to the sides of the 96-well plates to prevent the medium from evaporating. The suitable holes in the 96-well plates were selected as sample holes and control holes, and three groups of parallel holes were set, respectively. The cells in the logarithmic growth stage were digested with trypsin, and culture medium was added to homogenate the cells into a cell suspension with a density of 2.5 × 10^4^ cells/mL. The 200 *μ*L cell suspension was inoculated in each well. Then, the 96-well plates were placed in a wet incubator at 37°C with 5% CO_2_ for 24 hours. The next day, the 96-well plates were taken out, the original medium was sucked out, and an equal volume of PBS solution was added. Shaking gently, the mixture was sucked out and the whole process was repeated three times. The lectin medium with different concentrations of 100 *μ*L were added, and the medium containing 1‰ DMSO was added into the control well; then, the culture was continued for 24, 48, or 72 hours. We added 10 *μ*L CCK-8 solution to each well, then we incubated it in the incubator for 1 hour, and we detected OD_450_ with the enzyme marker. The inhibition rate of AAL on tumor cells was calculated according to the following formula (%).

(1) $$\begin{array}{c} \text{Cell inhibition rate }\left(\%\right)=\left[1-\frac{{\text{OD}}_{\text{samples}}}{{\text{OD}}_{\text{control}}}\right]\times 100\%. \end{array}$$

SPSS statistical analysis software was used to calculate half of the inhibitory concentration (IC_50_) of lectin on tumor cells.

### 2.5. Study on the Antitumor Molecular Mechanism of AAL

#### 2.5.1. RNA Sequencing

In order to further understand the molecular mechanism of AAL inhibiting the growth of tumor cells, we chose the A549 cells cultured with the initial concentration of 100 *μ*g/mL lectin for 72 hours as the experimental group, and we chose the A549 cells cultured without lectin for 72 hours as the blank control group. There were 3 replicates in the experimental group and the control group for RNA sequencing.

#### 2.5.2. mRNA Library Construction and Sequencing

Total RNA was extracted using the TRIzol Reagent (Invitrogen, CA, USA) following the manufacturer's procedure. The total RNA quantity and purity were analyzed using the 2100 Bioanalyzer and RNA 6000 Nano LabChip Kit (Agilent, CA, USA) with RIN number > 7.0. Approximately 10 *μ*g of total RNA representing a specific adipose type was subjected to isolate poly(A) mRNA with poly-T oligoattached magnetic beads (Invitrogen). Following purification, the mRNA is fragmented into small pieces using divalent cations under elevated temperature. Then, the cleaved RNA fragments were reverse transcribed to create the final cDNA library in accordance with the protocol for the mRNA-Seq sample preparation kit (Illumina, San Diego, USA); the average insert size for the paired-end libraries was 300 bp (±50 bp). And then we performed the paired-end sequencing on an Illumina Hiseq 4000 at LC Sciences, USA, following the vendor's recommended protocol.

#### 2.5.3. Bioinformatics Analysis

In this paper, we quantified gene expression abundance by calculating the FPKM value of the gene. We performed transcript abundance estimation and differentially expressed testing as follows: The mapped reads of each sample were assembled using StringTie. Then, all transcriptomes from Samples were merged to reconstruct a comprehensive transcriptome using perl scripts. After the final transcriptome was generated, StringTie and edgeR were used to estimate the expression levels of all transcripts. StringTie was used to estimate the expression level for mRNAs by calculating FPKM. The differentially expressed mRNAs and genes were selected with log2(fold change) > 1 or log2(fold change) < −1 and with statistical significance (*P* value < 0.05) by R package. The differentially expressed genes were screened when the inhibition rate of the A549 cells reached the maximum. The GO database and KEGG database were used for functional annotation and signal pathway enrichment analysis of the differentially expressed genes, and *P* < 0.05 was regarded as the significance threshold. According to the results of GO and KEGG functional annotation, tumor-related immune and apoptosis genes were screened out, and PPI analysis was performed to screen out key tumor factors. Relevant signal pathways were analyzed for the signal pathway gene network, so as to further search for tumor key genes.

### 2.6. The Regulatory Effect of Lectin on the Pulmonary Flora

#### 2.6.1. Sample Collection

We took A549 cells at their logarithmic growth phase, stained them with 0.4% trypan blue staining solution, counted viable cells under a microscope to ensure that the cell viability is greater than 95%, and adjusted the concentration to 2.5 × 10^6^ cells/mL with physiological saline. The mice were injected with 5 × 10^5^ A549 cells per mouse through the tail vein to establish a tumor-bearing mouse model. All the mice were male mice, and they were divided into 2 groups by a random number table: the treatment group and the control group, each with 8 mice. After 2 weeks, the gavage operation of the corresponding experimental group was completed. We gavaged mice with 50 mg/kg lectin or 0.2 mL normal saline daily for 14 days. Lung tissues of mice were weighed, and genomic DNA was extracted from the left lung.

#### 2.6.2. 16S Ribosomal RNA Gene Sequencing and Data Analysis

The V3-V4 region of the bacteria's 16S ribosomal RNA (rRNA) gene was amplified by PCR with barcode-indexed primers. Amplicons were then purified by gel extraction and were quantified using QuantiFluor-ST. After normalization, PCR amplicons were sequenced on an Illumina MiSeq platform (PE250). Alpha diversity was evaluated based on the following metrics: observed species and Shannon diversity index. A nonparametric two-sample *t*-test was used to compare the alpha diversity metrics between the control group and the treatment group.

## 3. Results and Discussion

### 3.1. Extraction of Lectin from *A. auriculate*

**Figure 1:**
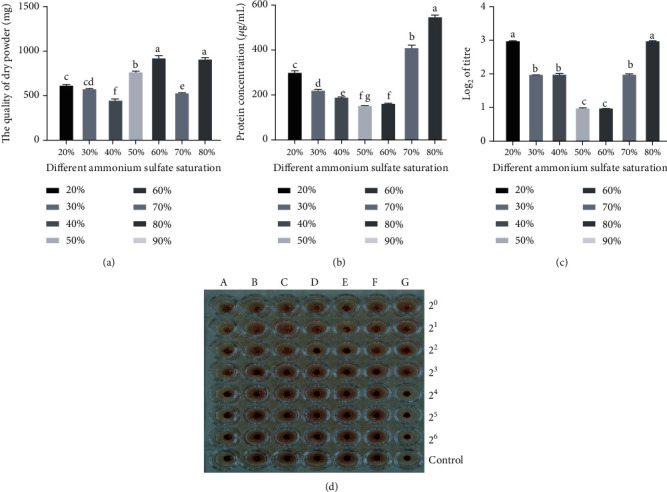
Effect of ammonium sulfate fractional precipitation on the extraction rate of lectin. Different ammonium sulfate saturations affect the quality of dry powder (a), protein concentration (b), and protein concentration (c). Hemagglutination activity of ammonium sulfate fractional precipitation (d) (A-G: crude extract precipitated by ammonium sulfate with a saturation of 20%-80%; 2^0^-2^6^: concentrations of lectin crude extract, respectively, in turn 1 mg/mL, 0.5 mg/mL, 0.25 mg/mL, 0.13 mg/mL, 0.06 mg/mL, 0.03 mg/mL, and 0.015 mg/mL. PBS was used as control).

After a preliminary analysis, we found that ammonium sulfate with 80% saturation was the best extraction method for the crude extract of AAL (Figure 1(a)), and the clotting activity of the crude extract separated by 20% and 80% saturation of ammonium sulfate was 2^3^, far higher than other extracts (Figures 1(c) and 1(d)). We also discovered that the crude extract protein concentration decreases firstly and then increased when the ammonium sulfate saturation increased, peaking at 80% saturation (Figures 1(b) and 1(d)). Considering the nature of the lectins, we determined that 80% saturation of ammonium sulfate is the best extracting method for crude extracting from *A. auriculate*.

### 3.2. Purification of Lectin from *A. auriculate*

**Figure 2:**
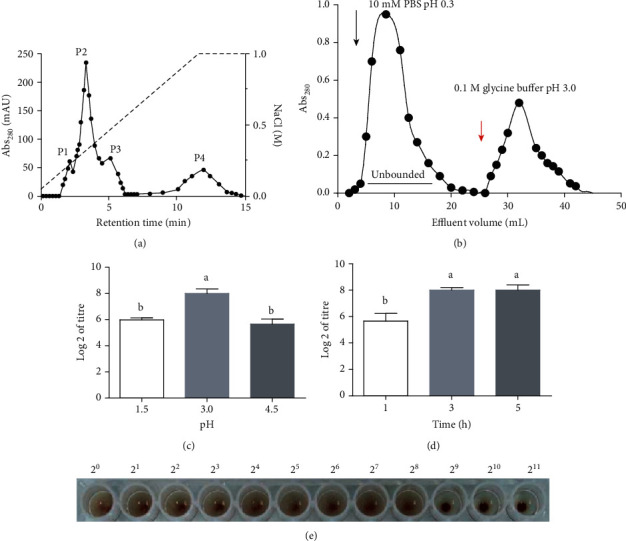
Processes and conditions for the purification of AAL. Ion exchange chromatograph of the crude extract of AAL (a). Affinity chromatograph of AAL (b). Effects of eluent conditions (c) and ultrafiltration time (d) on hemagglutination activity of AAL. Hemagglutination activity of AAL at different titres (e).

A HiTrap DEAE column was subjected to ion exchange chromatography, and four elution peaks P1, P2, P3, and P4 appeared at 280 nm at last. The elution components corresponding to each peak were collected, and the results showed that component P2 had the largest absorption peak (Figure 2(a)), and we finally identified its coagulation activity as 2^5^. Collected for freeze-drying, the proteins with specific binding capacity were bound to PSM-Sepharose by changing the pH of the system. There was a single elution peak of lectin isolated by affinity chromatography, which indicated that we got a single purity lectin through the extraction and purification steps (Figure 2(b)). In the process of affinity chromatography, elution conditions and ultrafiltration time were optimized, and hemagglutination activity was used as the evaluation criterion. When the coagulation activity of the eluent reached a maximum of 2^8^ at pH 3.0 and ultrafiltration time of 3 h (Figures 2(c) and 2(d)), the optimal hemagglutination activity of this result was shown (Figure 2(e)). The possible reason was that the pH of the eluent was too small, which affected the activity of lectin, while if the pH was too large, the lectin cannot be eluted completely.

Through the separation and purification and the optimization of the conditions, a pure AAL was obtained, and the purification multiple of AAL was 30.53 (Table 1). According to the formula, the extraction rate of AAL was 0.068%.

In this experiment, ammonium sulfate precipitation, HiTrap DEAE anion exchange chromatography, and PSM-Sepharose 4B affinity chromatography were used to separate and purify AAL with a molecular weight of 25 kDa from *A. auricula* for the first time. By SDS-PAGE detection, the lectin was found to be a single-subunit protein. The extraction conditions were optimized, with 80% ammonium sulfate precipitation and extraction, as well as eluent pH 3.0 and ultrafiltration time 3 h during affinity chromatography, were selected as the optimal extraction scheme of lectin, and the extraction rate was 0.068%. After three steps of separation and purification, a single lectin was obtained from *A. auricula*, and the purification factor was 30.53. In recent years, many researchers have discovered lectin in edible fungi. Tateno et al. isolated a 35 kDa tetramer lectin from sulfur bacteria by Sepharose 4B one-step affinity chromatography. Jiang used ammonium sulfate precipitation and GlcNAc-sepharose 6B affinity chromatography to purify AAL-2 with a molecular weight of 43.175 kDa from *Agrocybe cylindracea* [38].

### 3.3. Determination of Relative Molecular Weight of AAL

**Figure 3:**
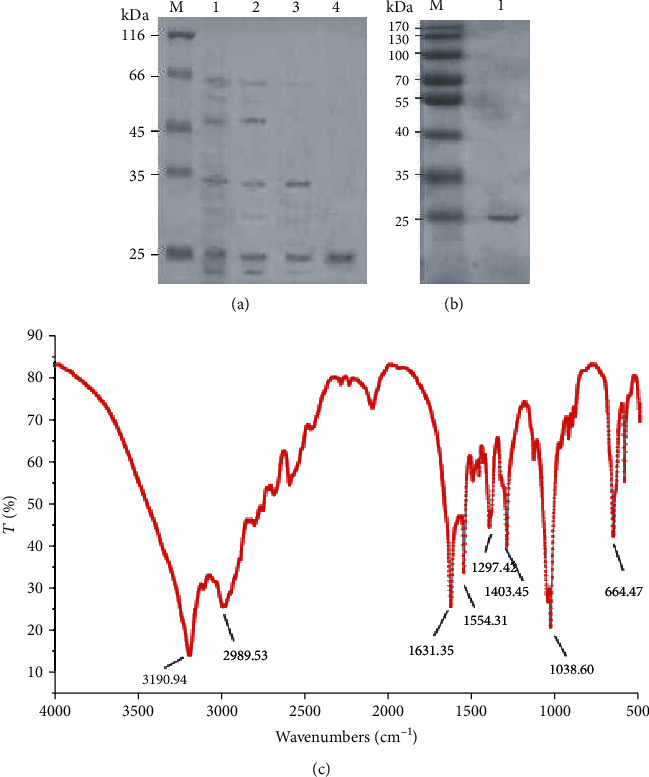
SDS-PAGE for the different periods of AAL and FT-IR detection. (a) Molecular weight detection of AAL by different extraction steps (M: standard molecular weight protein; 1: crude extraction components; 2: 80% ammonium sulfate precipitation component; 3: components of ion exchange chromatography; 4: affinity chromatography components). (b) Molecular weight of AAL was detected after *β*-mercaptoethanol treatment (M: standard molecular weight protein; 1: AAL after *β*-mercaptoethanol treatment). (c) FT-IR detection of AAL.

The molecular weight of lectin was detected by SDS-PAGE. After the purification process of ammonium sulfate precipitation, ion exchange chromatography, and affinity chromatography, a band with a molecular weight of 25 kDa was obtained (Figure 3(a)). After the AAL treatment by *β*-mercaptoethanol, the SDS-PAGE results were still a band of 25 kDa (Figure 3(b)). This suggested that AAL was a single subunit of glycoprotein.

Matrix-assisted laser desorption-ionization time-of-flight mass spectrometry (MALDI-TOF/TOF 5800) was used to analyze the relative molecular weight of AAL, and it was finally confirmed as 18913.22. The lectin protein was identified by a mass spectrometer (Applied Biosystems, MALDI-TOF/TOF 5800). Through UniProt database comparison, it was concluded that the lectin might contain four peptides, and the amino acid sequences were QIDAERK, TNHSVVTWNDK, RLNFTAGNPFPR, and VRELEQQVDSMTK, respectively. These sequences were not found in the existing *A. auricula* protein mass spectrometry library, so it was inferred to be a new protein in *A. auricula* and also determined to be a new lectin. The protein polypeptide sequencing machine was used to determine the amino acid sequence. Finally, the 13 amino acid sequences of the N-terminal were determined, and they are as follows: ITAPTTTSSAATE.

FT-IR was used to identify the binding mode of the oligosaccharide chain and the peptide chain of lectin. The infrared spectrum scanning range of lectin was from 400 cm^−1^ to 4000 cm^−1^, and the absorption peak near 3190.94 cm^−1^ was the stretching vibration peak of the polysaccharide O-H bond. A wide absorption peak near 2989.53 cm^−1^ was the characteristic absorption peak of the C-H bond bending and vibration. The strong absorption peaks around 1631.35 cm^−1^ and 1554.31 cm^−1^ are the C=O bond stretching vibration peak and the N-H bond bending vibration peak on the amide. The absorption peak near 1403.45 cm^−1^ was characteristic of polysaccharide. The strong absorption peak near 1297.41 cm^−1^ was the stretching vibration absorption peak of the C-N bond on the amine. The strong absorption peak near 1038.60 cm^−1^was either the C-O bond, the C-C bond stretching vibration, or the C-OH bond bending vibration of the polysaccharide chain. The strong absorption peak near 664.67 cm^−1^ is the bending vibration outside the C-H bond plane on the benzene ring (Figure 3(c)).

### 3.4. Analysis of Physicochemical Properties of Lectin from *A. auricula*

#### 3.4.1. Effects of Temperature and pH on Hemagglutination Activity

A solution of 1 mg lectin was prepared and incubated in a water bath at 20°C-100°C for 40 min. The hemagglutination activity of the lectin was observed to remain unchanged within 50°C but gradually declined when higher than 50°C. At 90°C, the hemagglutination activity completely disappeared, indicating that the lectin was a temperature-sensitive glycoprotein. The lectin solution was incubated in a constant-temperature water bath at 50°C for 10-100 min, and the maximum hemagglutination activity was maintained within 40 min. With the increase of time, the hemagglutination activity decreased, and the hemagglutination activity remained unchanged after 80 min. The lectin was dissolved in PBS at different pH levels, and its ability to agglutinate rabbit blood was tested. At pH 2, there was no hemagglutination activity. With the increase of pH, the hemagglutination activity of AAL increased gradually. At pH 7-8, the hemagglutination activity remained stable. While at pH 9, the hemagglutination activity decreased significantly. When the pH reached 10, the hemagglutination activity was completely lost, indicating that the lectin was an acid–base-dependent protein.

#### 3.4.2. Sugar Specificity of AAL

When joining all kinds of sugars (derivative) and proteins in the AAL solution, respectively, to verify the inhibitory activity of blood coagulation, the results showed that glucose, galactose, mannose, rhamnose, and xylose monosaccharide sugar do not suppress AAL activity of blood agglutination. When N-acetyl galactosamine and sialic acid derivatives such as sugar were added to AAL, there was no activity of blood agglutination inhibition. The hemagglutination activity of AAL was inhibited only when porcine mucosal protein was added, and the minimum inhibitory concentration of lectin was 5 *μ*g/mL, which indicated that the AAL did not have a specific recognition effect on monosaccharides and sugar derivatives, but had a specific recognition effect on mucoprotein (Table 2).

#### 3.4.3. Protein Quantification and Sugar Content of AAL

Using the BCA protein quantitative kits, we detected the protein concentrations of lectins using the linear regression equation *Y* = 0.7968 + 0.1164*X*, with the correlation coefficient *R*^2^ > 0.99; the results were believable. The enzyme standard instrument had a 562 nm absorbance value and a sample hole measuring absorbance value of 0.283, according to the standard curve obtained by AAL protein concentration which was 209 *μ*g/mL. According to the phenol-sulfuric acid method for determining the sugar content of AAL, the linear regression equation was Y = 0.1803 + 0.0216X, and the correlation coefficient was *R*^2^ > 0.99, which indicate that the results were credible. The enzyme standard instrument had a 490 nm absorbance value and a sample hole measuring absorbance value of 0.296, according to the standard curve to calculate AAL sugar concentration which was 1.52 mg/mL; the polysaccharide content was 10.3%.

Previous studies have found that most lectins extracted from edible fungi are single-subunit proteins, and all of them show agglutination activity against erythrocyte. For example, Lin et al. found that lectin from edible fungi could agglutinate not only human erythrocytes but also rabbit erythrocytes, with stronger hemagglutination activity against rabbit erythrocytes [39]. Guo et al. studied the clotting activity of various edible fungi on chicken blood erythrocytes, and the results showed that most of them had blood clotting function [40]. In this experiment, lectin had agglutinating activity on rabbit blood, and the maximum titer was 2^8^. Carbohydrate recognition domain (CRD) is used for the binding of lectin and for recognizing the position of the sugar chain. The sugar or protein specifically binding lectin from different sources is different. Osterne et al. obtained the specific lectin of *α*-cymene-D-mannoside from honeysuckle seeds which showed anti-inflammatory and cytotoxic activities [41]. Li et al. isolated and purified N-acetyl galactosamine/galactose-specific agglutinin from marine invertebrate mussels [42]. Wu et al. isolated trehalose-specific lectin from oyster sperm and demonstrated its structural model [43]. The results of the sugar inhibition of lectin showed that monosaccharides and sugar derivatives had no specific binding activity, while pig gastric mucosa protein (PSM) had an inhibitory effect on its coagulation activity. The minimum inhibition concentration was 5 *μ*g/mL, indicating that lectin had specific binding to pig gastric mucosa protein. In addition, the coagulation activity of lectin decreased or was even lost under high temperatures and acidic and alkaline conditions, indicating that AAL is a kind of temperature-sensitive, acid–base-dependent glycoprotein. The protein quantitative results showed that the protein concentration of lectin was 209 *μ*g/mL, and the sugar content of lectin detected by the phenol-sulfuric acid method was 10.3%. This indicates that lectin was a kind of complex glycoprotein with more sugar chains. Fourier transform infrared spectroscopy (FT-IR) and *β*-elimination reaction of the *A. auricula* lectin structure were analyzed, and the results show that the linkage between the lectin glycosyl chain and the polypeptide is an N-glucoside bond. A similar research also verified the lectin of the sugar chain with the connecting way of peptides. Yao et al. extracted lectins, and the structure of the lectins was analyzed by FT-IR, *β*-elimination reaction, and spectroscopy. The results show that the lectins are connected to the sugar and protein in the form of an O-glycosidic bond, and the secondary structure was mainly an *α*-helix and a random coil [44]. Cui et al. obtained a glycoprotein from the purification of *Grifola frondosa mycelia*, and they identified the glycoprotein by FT-IR, NMR, and *β*-elimination reactions as being attached to the sugar and protein by O-glucoside bonds. The roundabout spectrum results showed that the glycoprotein was mainly stable in the secondary structure of *β*-folding [45].

### 3.5. Study on the Antitumor Activity of AAL

#### 3.5.1. Inhibition of the Proliferation of Lung Cancer Cell A549

This experiment set different concentrations of AAL for an early screening of A549 cells. The results showed that compared with the negative control group, when the concentration of lectins was 250 *μ*g/mL, A549 cells showed a trend of apoptosis and the growth of A549 cells was significantly suppressed. When the concentration of the lectin was 100-200 *μ*g/mL, the number of cells decreased and cell bubbles appeared. When the concentration of the lectin was 50 *μ*g/mL, the cell morphology of A549 cells did not change significantly. It was only observed that the cells began to gather and the cell volume increased, so it was judged that the cells began to appear together with the bubbles. Therefore, 100 *μ*g/mL was chosen as the initial concentration of lectin to inhibit the proliferation of A549 cells.

**Figure 4:**
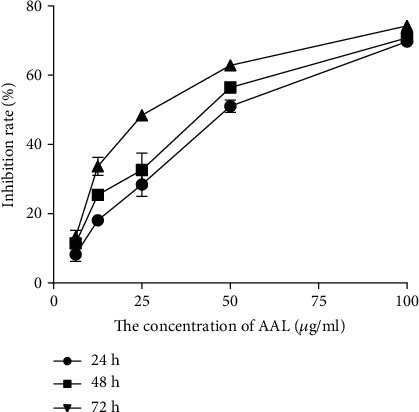
Inhibitory curve of AAL on A549.

In a compound sieve experiment, we chose 100 *μ*g/mL of AAL as the initial concentration, and the concentration gradient was diluted (100 *μ*g/mL, 50 *μ*g/mL, 25 *μ*g/mL, 12.5 *μ*g/mL, and 6.25 *μ*g/mL). A549 cells were treated for 24 h, 48 h, and 72 h under different lectin concentrations, then we used the CCK-8 Kits to test the lectin effect on the growth inhibition of A549 cells. The results showed that the growth of A549 cell inhibition was concentration and time dependent, the degree of A549 cell proliferation inhibition gradually strengthens with the increase of the concentration of lectins. And at the same concentration but processed at different times, the degrees of inhibition of A549 cells were different. When the concentration of AAL was 100 *μ*g/mL and AAL was cultured for 72 hours, the inhibition rate of A549 cells reached a maximum of 74% (Figure 4).

#### 3.5.2. Half-Inhibitory Concentration (IC_50_) of AAL

The half-inhibitory concentration (IC_50_) was the concentration at which the drug restricts the growth of half of the tumor cells. It is the standard to measure the effectiveness of the drug on the tumor cells, and it is used by new drugs to evaluate the targeting of cancer cells. Here, we studied the effect of *A. auricula* lectin on lung A549 cancer cells at 24 h, 48 h, and 72 h. Tumor cell proliferation inhibition rate was concentration and time dependent on AAL, and through the CCK-8 kit, we detected the proliferation of A549 cells. The IC_50_ was 50.17 ± 2.69 *μ*g/mL for 24 h, 41.29 ± 2.22 *μ*g/mL for 48 h, and 28.19 ± 1.92 *μ*g/mL for 72 h. The results show that AAL had a good inhibition effect on the proliferation of A549 cells.

Cancer is a serious threat to human health. Previous studies found that there were 18.1 million new cancer cases and 9.6 million cancer deaths worldwide in 2018, and it is estimated that nearly half of all new cancer cases and more than half of all cancer deaths occurred in Asia; thus, the incidence of cancer is very serious. The incidence of cancer in China accounts for about 22% of the world's total, and the number of cancer cases is the highest in the world [29]. In recent years, lung cancer (LC) is still one of the most common malignant tumors in human beings, and the incidence and mortality of LC are increasing year by year [46, 47]. Currently, the drugs used to treat tumors are time-consuming, damaging, and expensive. Therefore, the pursuit of green, safe, and targeted edible antitumor drugs has become the top priority. Lectins found in terrestrial plants and edible fungi have antitumor activities, such as mulberry leaf lectins' sensitization to McF-7 by activating the P38 MAPK protein kinase to inhibit the signal transduction pathway mediated by fibrin [48, 49]. Tea mushroom lectin is easily absorbed by the lungs of mice, preventing 4T1 breast cancer cells from transferring to the lungs and preventing mice from suffering from secondary infection [50]. Ruthenate lectin acts as a molecular switch to control the apoptosis and autophagy of A549 cells. At the same time, lectin in the lymphatic fluid of marine mollusk shellfish also has a good inhibitory effect on tumor proliferation [51]. For example, the marine mussel lectin has the ability to recognize the sugar on the Gb3 receptor of tumor cells and thus inhibit the proliferation of McF-7 in breast cancer cells.

### 3.6. Study on the Antitumor Molecular Mechanism of Lectin

In order to study the antitumor molecular mechanism of AAL, we conducted RNA sequencing. The differential genes were screened when the inhibition rate of A549 cells reached the maximum. By RNA sequencing, different expression genes in A549 treated with lectin and without lectin were counted. A total of 350 differentially expressed genes were found, among which 194 were upregulated and 156 were downregulated.

**Figure 5:**
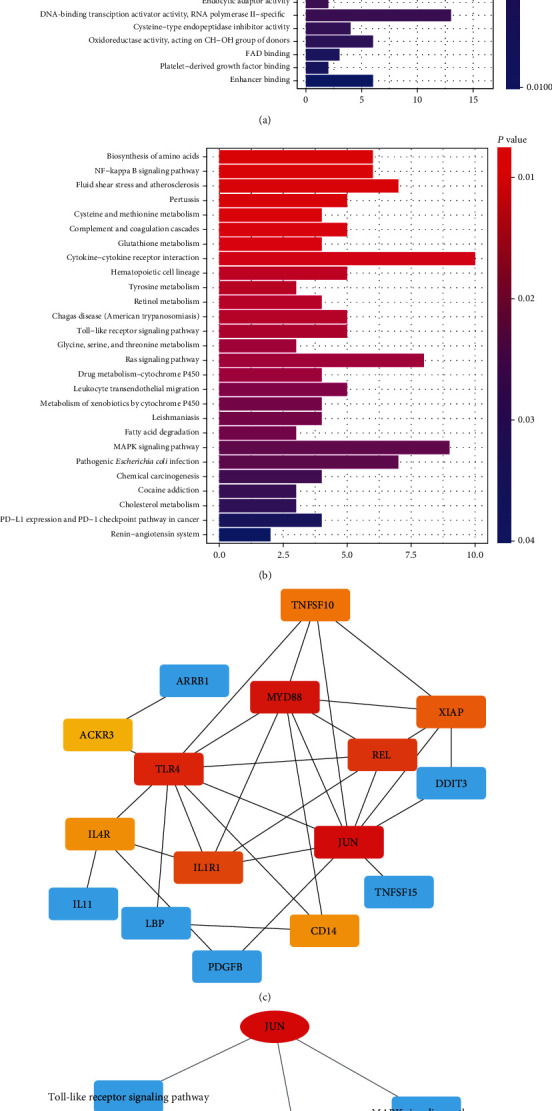
Antitumor molecular mechanism of lectin. GO annotation of the different expression genes (a). Signal pathway enrichment of the different expression genes (b). PPI plot of genes related to immunity and apoptosis (c). Network analysis of key genes and related signaling pathways (d).

Through the GO and KEGG functional annotation and signal pathway enrichment analysis of the differentially expressed genes, many tumor-related genes and signaling pathways have been found, such as the NF-kappa B (NF-*κ*B) signaling pathway, the Toll-like receptor signaling pathway, the PD-L1 expression and PD-1 checkpoint pathway in cancer, and the MAPK signaling pathway (Figures 5(a) and 5(b)).

### 3.7. Screening the Key Factors of Lectin Action on the A549 Cells

According to the results of GO and KEGG functional annotation, tumor-related immune and apoptosis genes were screened out, and PPI analysis was performed to screen out key tumor factors (Figure 5(c)). Relevant signaling pathways were analyzed for the signaling pathway gene network, so as to further search for tumor key genes (Figure 5(d)). The results showed that *JUN*, *TLR4*, and *MYD88* were the key regulatory factors.

By functional annotation of differentially expressed genes, it was found that GO mainly concentrated in endopeptidase regulator activity, endopeptidase inhibitor activity, enzyme inhibitor activity, and peptide binding and peptidase inhibitor activity. The KEGG pathway was mainly enriched in the NF-*κ*B signaling pathway, the Toll-like receptor signaling pathway, the PD-L1 expression and PD-1 checkpoint pathway in cancer, and the MAPK signaling pathway. As we all know, NF-*κ*B signaling plays an important role in inflammatory pathways and apoptosis [52]. Activation of the NF-*κ*B signaling pathway is an important tumor-inducing modulator in many cancers such as cervical cancer, enabling tumor cells to evade apoptotic cell cycle checkpoints [53]. In addition, it is said that NF-*κ*B plays a critical role in the inflammatory pathways of malignancies, such as activating NF-*κ*B that encourages cell proliferation, survival, and angiogenesis [54]. Bacteria may disrupt the cell cycle by toxin production, resulting in cell growth with alterations in protein expression that control DNA repair, cell division, and apoptosis [55]. Furthermore, bacteria may alter the host immune response against malignant cells, and an association between microbiota composition and clinical immunotherapy response has recently been shown. Studies in animal models indicate that microbiota modulate the sensitivity of solid cancers to immune checkpoint inhibitors (ICIs), mainly cytotoxic T-lymphocyte-associated protein 4 (CTLA4) and PD-1/PD-L1 [56]. As we all know, lectin has an effect on lung microflora [57]. So, lectin may affect the occurrence and development of lung cancer by influencing the microflora. The TLR signaling pathway consists of two subpathways: the *MYD88*-dependent pathway and the *MYD88*-independent pathway. There is increasing evidence that TLRs are an important regulator of tumor biology. The regulation of the TLR signaling pathway plays an important role in the occurrence or progression of tumors [58]. Grimmig et al. reported that the TLR signaling pathway promoted proliferation of pancreatic cancer cells [59]. Besides, *TLR3* has been found to stimulate cancer cell survival, proliferation, and progression in breasts [60], pharynx [61], and head and neck [62]. For lung cancer, the TLR pathway may play a key role in tumorigenesis and progression, especially in non-small-cell lung cancer (NSCLC) [63–65]. Therefore, some genetic variants in genes of this pathway may have a predictive value as a clinically potential biomarker for outcomes of NSCLC. Moreover, through the correlation analysis between PPI and genes, we found that *JUN*, *TLR4*, and *MYD88*, the three main regulatory genes of lectin, were all highly expressed in the tumors, and the expression of these three genes were inhibited after treatment by AAL.

### 3.8. Lectin Could Regulate the Pulmonary Flora

Compared with the control group (0.355 ± 0.041 g), the weight of the lung tissue in the lectin treatment group (0.242 ± 0.026 g) was significantly reduced, which proved that lectin could inhibit the growth of lung cancer cells in vivo. In sequencing analysis of respiratory flora of tumor-bearing mice after lectin treatment, 259 bacterial colonization species were detected in the control group and 538 bacterial colonization species were significantly increased in the lectin treatment group. Among them, 62 species of bacteria were common strains. The bacterial analysis results showed that compared with the control group, the lung microflora structure of the lectin treatment group was significantly different (*P* < 0.05), the bacterial structure of each phylum in the lectin treatment group was more reasonable, and the colonization ability of the normal microflora was improved, indicating that lectin treatment could significantly improve the bacterial diversity characteristics of the tumor-bearing mouse model (Table 3).

In recent years, with the in-depth development of molecular biology research technology, it has become clear that lung tissue is not always in a balanced state. Studies have shown that inhaled upper respiratory tract secretions can bring part of the oropharyngeal microbial community into the lung tissue; at the same time, the host activates the defense mechanism, which can effectively eliminate microbes and achieve the purpose of blocking infection. The manifestations of changes in the respiratory microecosystem are complex and diverse, and may eventually lead to local or systemic bacterial infections. However, in the course of lung cancer, the changes in the respiratory tract microflora have not been clearly understood.

In this study, by detecting changes in the respiratory tract flora of non-small-cell lung cancer-bearing mice, the effect of lectin treatment on the respiratory tract flora of A549 tumor-bearing mice was explored. The results of the study showed that *Firmicutes*, *Actinomycetes*, *Bacteroides*, *Proteobacteria*, and *Fusobacteria* in the respiratory tract have certain colonization in the respiratory tract of tumor-bearing mice. At the same time, compared with the control group, there are obvious differences in the structure of the lung flora in the lectin treatment group. The bacterial structure of each phyla in the lectin treatment group is more reasonable, indicating that lectin treatment can significantly increase the diversity of the lung flora of A549 tumor-bearing mice, promote the balance of respiratory flora, and enhance the body's biobarrier effect, reducing the colonization of pathogenic bacteria.

## 4. Conclusion

In this study, lectin was isolated from *A. auricula* by affinity chromatography and named AAL. Through 80% (NH_4_)_2_SO_4_ precipitation, HiTrap DEAE anion exchange chromatography, and PSM-Sepharose 4B affinity chromatography (eluent pH 3.0, ultrafiltration time 3 h), the purification process of AAL was obtained, and the optimal extraction rate was 0.068%. AAL is a monosubunit protein with a molecular weight of 25 kDa. The relative molecular weight of lectin AAL as determined by MALDI-TOF/TOF is 18913.22. The N-terminal sequence was detected as ITAPTTTSSAATE. After protein identification and comparison with the UniProt database, it was concluded that the lectin contained 4 peptides, whose amino acid sequences were QIDAERK, TNHSVVTWNDK, RLNFTAGNPFPR, and VRELEQQVDSMTK. The connection between the oligosaccharide and polypeptide of AAL was the N-glucoside bond. Physical and chemical property analyses showed that AAL was a temperature-sensitive and acidic/alkaline-dependent glycoprotein. Additionally, the anticancer experiment manifested that AAL inhibited the proliferation of A549 and the IC_50_ value was 28.19 ± 1.92 *μ*g/mL. RNA sequencing and TCGA dataset analyses detected that AAL may regulate the expression of *JUN*, *TLR4*, and *MYD88* to suppress tumor proliferation. We finally found a lectin that could regulate the pulmonary flora.

## Figures and Tables

**Table 1:** Statistics of AAL extraction by different extraction methods.

Separation method	Total protein (mg)	Total coagulation activity (HU)	Specific activity (HU·mg-1)	Purification fold	Recovery rate (%)
Crude	620.00	3200	5.16	1	100
(NH_4_)_2_SO_4_ precipitation	279.00	2840	10.18	1.79	88
HiTrap DEAE	36.20	1280	35.36	6.85	40
PSM-Sepharose 4B	6.50	1024	157.54	30.53	32

**Table 2:** The carbohydrate specificity of AAL.

Sugars (derivatives) and proteins	Minimum inhibition concentration (mM/*μ*g·mL^-1)^
Glc	N.i.^1^
Gal	N.i.
Man	N.i.
Rha	N.i.
Xyl	N.i.
GlcNAc	N.i.
PSM	5
SA	N.i.

^1^No inhibition.

**Table 3:** The abundance distribution of bacteria.

Classification	Lectin treatment group	Control group
Firmicutes	3697	1172
Bacteroidetes	2321	108
Actinobacteria	1846	54
Proteobacteria	1211	475
Cyanobacteria	469	16
Chloroflexi	357	16
Acidobacteria	216	44
Fusobacteria	16	37
Others	315	43

## Data Availability

The data underlying the findings of this article will be shared by the corresponding authors upon reasonable request.

## Additional Points

*Sample Availability*. Samples of the compounds are available from the authors.
